# The Feeding of Infants and Children. II.—In Disease

**Published:** 1892-11-19

**Authors:** 


### PADDINGTON GREEN CHILDREN'S HOSPITAL.

### The Feeding of Infants and Children.

### II.—In Disease.

There are some infants for whom, owing to idiosyncrasy rather than disease, no general rules of diet can be laid down. In such cases it may be necessary to substitute for the breast or cow's milk, condensed milk, peptonised milk, &c., but whatever substitute is employed it should be regarded as temporary, and efforts made to bring the patient back as soon as possible to normal diet. In this paper we shall first indicate generally the food materials employed, and the methods of their preparation, and then refer to the diet employed in certain diseases. The accompanying medicinal treatment will not be discussed.

If the boiled cow's milk and barley-water are unsuited to an infant, as shown by wasting, persistent sickness with curdy vomit, constipation, or diarrhœa, then lime-water is added in the proportion of two tablespoonfuls to each half bottle of milk, or fifteen to thirty drops of liquor calcis saccharatus, or koumiss may be used, or the milk may be peptonised. The method of peptonising the milk is as follows: To one pint of fresh milk and a quarter of a pint of cold water is added one of Fairchild's peptonising powders. The mixture is then put in a jar, which is set in a basin containing water as hot as the hand can bear, and allowed to stand for twenty minutes, the jar being occasionally shaken. The milk is then removed, and rapidly brought to the boil, so as to stop the peptonising process, after which it is ready for use. In other cases, where only a small quantity of milk is tolerated, raw meat juice is employed with very good results. To make it, a quarter of a pound of the best beef steak is first passed through the mincing machine, and then pounded to a paste. To this is added a quarter of a pint of cold water, and the whole is allowed to stand for an hour, after which it is strained through muslin. To the juice thus obtained, salt or sugar may be added according to taste, and one ounce or more may be given every four hours. Infants will take it readily if it is slightly warmed, sweetened, and mixed with milk. It is to be noted that small quantities only should be made at a time, as the meat juice is very liable to decompose on keeping. In the case of older children, raw lean meat can be substituted for the meat juice. It is made by scraping with a knife a piece of lean beef, and the pulp thus obtained is spread between thin slices of bread. After a few days of this diet, with or without small quantities of milk, the stomach will often be found to have recovered its tone, and the normal diet can be gradually returned to, while in some delicate infants the mixture of meat juice and milk may be given for months with advantage. In other cases it is found advisable to give the digestive system as complete a rest as possible for twelve hours, or even longer. For this purpose "bread jelly" is found useful. It is made according to the directions given by Dr. Cheadle. A thick slice of stale bread is placed in a basin of cold water, and allowed to soak for eight hours. The pulpy mass thus formed, after the water is squeezed out of it, is put in one and a-half pints of fresh water and gently boiled for an hour and a-half. The thick gruel thus made is strained, rubbed through a fine hair sieve, and allowed to grow cold, when it forms a jelly-like mass. This jelly, diluted with twelve parts of water, and slightly sweetened, can be given either in the feeding bottle or with a spoon. The preparation is especially bland and unirritating, but should be prepared twice a day, as it does not keep well. To it can be added milk in gradually-increasing proportions as the stomach improves.

Another method of resting the stomach is by means of rectal feeding. Nutrient enemata must be small—half-an-ounce for an infant, two to three ounces for an older child—and they must not be continued for more than twenty-four or forty-eight hours, after which time they are not usually retained. Peptonised preparations of milk or meat juice are specially suitable, and brandy, Valentine's meat juice, and beef tea are also used. It is usual to clear out the rectum thoroughly with a warm water enema as a preliminary. The nutrient enema is then warmed, and slowly introduced by means of a glass syringe, and it can be repeated every three or four hours as required. As a nutrient enema is believed to induce reflexly a certain amount of gastric secretion, a few spoonfuls of barley water, or weak milk and water are administered by the mouth at the same time. When there is a difficulty in swallowing, food is introduced directly into the stomach through a rubber tube passed along the floor of the nose and down the œsophagus. To the outer extremity of this tube a small glass funnel is attached. Before inserting the tube, the extremity is dipped in glycerine to facilitate its passage. There is apt to be a vigorous protest on the part of the patient at first, but toleration is soon established. In some cases of extreme weakness, where even sucking requires too great an effort, this method of feeding will disturb the child less than any other. Care must be taken to clean out the rubber tube after use by syringing hot water through it, so as to prevent any particles of food adhering to it.

We now pass on to describe the diet in particular diseases. In typhoid fever, the patient on admission is ordered plain cow's milk as the sole diet, the amount varying with the age of the child; for example, two pints for a child five years old. In some cases no other food will be given during the acute stage, but if the appetite is good the amount may be increased to three pints. The milk is administered at regular intervals, every two or three hours during the day, and every four hours during the night, and plain water or barley water is allowed between meals, should the patient desire it. If sickness is present, or flatulence, or pain after food, barley water or lime water is added in the proportion of two or three parts of milk to one of the diluent. If there are undigested particles of milk in the motions, or if diarrhœa is persistent, the milk is peptonised, and may also be curtailed in amount. If some additional nourishment is judged necessary, beef-tea, or raw meat juice (two to four ounces daily), or Valentine's meat juice (one teaspoonful every four hours), or port wine, or brandy is given. Beef-tea is used sparingly, seldom more than ten ounces per diem, and is avoided if there is much diarrhœa present. In some cases, where diarrhœa is excessive with very offensive motions, it is found useful to limit the patient entirely for twelve or twenty-four hours to bread jelly and barley water, with brandy. In the case of an acute wasting disease like typhoid, brandy is reckoned a valuable article of diet. Extreme care is also taken with the feeding in the convalescent stage. The following may be taken as an illustrative dietary where the progress of the patient is uninterrupted.

A. B., aged 5 years. Typhoid fever.—April (acute stage): Peptonised milk, two pints; barley water, one pint; brandy, ℥ij every four hours. May 1st (temperature normal): Peptonised milk, two and a-half pints; barley water, half pint. May 7th: Plain milk, two pints; beef tea, half pint; barley water, half pint. May 10th: Plain milk, rennet, chicken tea. May 12th: Add custard pudding. May 14th: Add fish, or chicken panada. May 16th: Add thin bread and butter without crust; boiled egg. May 21st: Add boiled mutton; cream, two ounces.

The object is to make the diet as varied, nourishing, and digestible as possible, but as a rule no solid food is allowed until ten or fourteen days after the temperature has reached normal. The rennet mentioned in the list is added to allay the child's craving for something solid. If the convalescent stage is interrupted, as by sickness or other internal disorder (not relieved by an enema, and accompanied with a rise in temperature), the patient is at once put on milk and barley water for a few days, whatever stage in the convalescent dietary may have been reached. If the symptoms pass off, the plan of progressive diet is resumed exactly the same as after the acute stage, and not at the point where it was interrupted.

For the ordinary diarrhœa of children, due to some indigestible and irritating food, a smart purge and plain milk food is usually sufficient. The acute summer diarrhœa of children, with its accompanying sickness, collapse, and rapid wasting, requires very energetic treatment. Food must be administered in very small quantities and frequently, every hour or half-hour, and stimulants in the form of brandy or champagne are given freely. Small pieces of ice to suck may relieve the sickness, and the food is often better tolerated when given cold. The disease being usually associated with fermentation of milk foods, these must often be stopped entirely, and meat juice well diluted, or meat pulp substituted for a time. In cases with persistent sickness, nutrient enemata are found useful. Diluent drinks, such as barley-water, are given freely. During the convalescent stage the utmost caution is exercised so as not to tax the exhausted digestive powers more than possible. For example, peptonised milk is followed by milk and lime-water, then milk and barley-water, and finally plain milk. There are many cases of tubercular diarrhœa where a most carefully regulated diet of milk, milk puddings, and eggs has no effect in checking the disease. In such cases a meat diet is substituted; for young children beef pulp on thin slices of bread, and for older children boiled beef or mutton, with bread, and claret (two to four ounces daily). As a rule the patients will enjoy this diet, and the rapid cessation of the diarrhœa is often very striking. As the patient's condition improves, the ordinary articles of food are gradually resumed. The chronic constipation of childhood is often due to too fluid a diet, and the judicious addition of Mellin's food, coarse oatmeal porridge, or brown bread will relieve the condition. In rickets or scurvy rickets the use of artificial or preserved foods is forbidden, and fresh cow's milk, cream, raw meat juice, the juice of oranges or lemons, and cod-liver oil with malt are given freely. The smearing of cod-liver oil on the abdomen is not practised. In acute nephritis milk is the staple diet, and with this may be combined simple puddings and boiled bread and milk; during the convalescent stage, and in chronic nephritis, no butchers' meat is given. In surgical cases requiring operation, the patient is prepared for a few days by a plain diet, and on the day of operation a meal of bread and milk is given not less than four hours before the administration of the anæsthetic. For the sickness after chloroform small pieces of ice are allowed, or sips of hot water, and nourishment is administered sparingly in the shape of brandy and water, or Valentine's meat juice, well diluted. The effect of the anæsthetic will usually have passed off within twelve or twenty-four hours, when milk, or milk and egg are given.

